# The mRNA content of plasma extracellular vesicles provides a window into molecular processes in the brain during cerebral malaria

**DOI:** 10.1126/sciadv.adl2256

**Published:** 2024-08-16

**Authors:** Mwikali Kioko, Shaban Mwangi, Alena Pance, Lynette Isabella Ochola-Oyier, Symon Kariuki, Charles Newton, Philip Bejon, Julian C. Rayner, Abdirahman I. Abdi

**Affiliations:** ^1^ Bioscience Department, KEMRI-Wellcome Trust Research Programme, Kilifi, Kenya.; ^2^ Open University, Milton Keynes, UK.; ^3^ Pathogens and Microbes Programme, Wellcome Sanger Institute, Cambridge, UK.; ^4^ School of Life and Medical Science, University of Hertfordshire, Hatfield, UK.; ^5^ Centre for Tropical Medicine and Global Health, Nuffield Department of Medicine, University of Oxford, Oxford, UK.; ^6^ Department of Psychiatry, University of Oxford, Oxford, UK.; ^7^ Cambridge Institute of Medical Research, University of Cambridge, Cambridge, UK.; ^8^ Pwani University Biosciences Research Centre, Pwani University, Kilifi, Kenya.

## Abstract

The impact of cerebral malaria on the transcriptional profiles of cerebral tissues is difficult to study using noninvasive approaches. We isolated plasma extracellular vesicles (EVs) from patients with cerebral malaria and community controls and sequenced their mRNA content. Deconvolution analysis revealed that EVs from cerebral malaria are enriched in transcripts of brain origin. We ordered the patients with cerebral malaria based on their EV-transcriptional profiles from cross-sectionally collected samples and inferred disease trajectory while using healthy community controls as a starting point. We found that neuronal transcripts in plasma EVs decreased with disease trajectory, whereas transcripts from glial, endothelial, and immune cells increased. Disease trajectory correlated positively with severity indicators like death and was associated with increased VEGFA-VEGFR and glutamatergic signaling, as well as platelet and neutrophil activation. These data suggest that brain tissue responses in cerebral malaria can be studied noninvasively using EVs circulating in peripheral blood.

## Teaser

The impact of malaria on brain tissues can be inferred by analyzing the biological cargo of circulating extracellular vesicles.

## INTRODUCTION

Cerebral malaria is an encephalopathy caused by *Plasmodium falciparum* infection (*1*, *2*) and was defined clinically as a coma in the presence of peripheral blood parasitemia and not directly attributable to other causes such as hypoglycemia, convulsion, and meningitis (*3*). Sequestration of parasitized erythrocytes in the brain microvascular system is a key mechanism leading to neurological impairment and the driver of disease severity in cerebral malaria (*2*). However, the impact of *P. falciparum* on cerebral tissues can only be studied directly during postmortem evaluation (*4*).

This limitation has motivated the search for noninvasive ways of accurately diagnosing cerebral malaria. The retina comprises brain-like tissues and shows parasite sequestration and pathology comparable to that in canonical brain tissues (*5*–*7*), but unlike the brain, the retina can be directly visualized; thus, sequestration-associated retinal pathology can be visualized clinically via noninvasive ophthalmological techniques (*8*). These approaches have revealed a retinal pathology, termed “malaria retinopathy,” that can be used as a surrogate marker for parasite sequestration in the brain (*9*–*12*). While studies of adults found no clear association between cerebral malaria and retinopathy (*12*, *13*), there are clear associations in children, among whom patients with retinopathy-positive cerebral malaria (CM-R^+^) are considered the “true” cases of *P. falciparum*–induced cerebral pathology and those with retinopathy-negative cerebral malaria (CM-R^−^) are suspected of having either an encephalopathy caused by other etiologies with incidental *P. falciparum* infection (*4*, *9*, *14*) or a less severe variant of cerebral malaria (*15*, *16*). Retinopathy was found to be highly sensitive (87 to 100%) and specific (75 to 87%) for pediatric cerebral malaria caused by *P. falciparum* (*17*), but it provides no information on the molecular mechanisms of cerebral malaria pathogenesis.

We propose a complementary noninvasive approach to studying cerebral malaria by analyzing extracellular vesicles (EVs) circulating in the blood. EVs are nanosized molecules secreted by all cells into biological fluids, are surrounded by a limiting phospholipid membrane (*18*), and have been linked to malaria pathogenesis (*19*). They contain biological cargo such as RNA, lipids, and proteins, which reflect the metabolic and physiological status of the parent cells or tissues. EVs can also cross tissue-blood barriers and circulate in biofluids without diluting their contents (*20*, *21*). This makes EVs attractive noninvasive tools for studying the pathology of diseases affecting inaccessible tissues, such as cerebral tissues (*18*), in contrast to circulating immune cells that provide information limited to the immune compartment (*21*, *22*).

The dichotomization of patients based on clinical parameters such as retinopathy often ignores the continuum nature of disease progression and fails to capture the within-category heterogeneity. In addition, binary outcome analysis provides static average profiles of the groups and not the trend of molecular alteration that occurs as the disease progresses. Researchers in other fields have used trajectory inference methods, such as manifold learning, to reconstruct disease progression models from cross-sectional gene expression data (*23*, *24*). Trajectory inference algorithms work under the premise that gene expression data from each sample represents an instance of a pathological state, and patients are ordered along a trajectory of pathological progression based on similarity in their gene expression profiles (*25*). Here, we apply manifold learning to construct a trajectory representing the progression of the pathophysiological states in cerebral malaria using plasma EV transcriptomes from cross-sectional samples obtained from patients with CM-R^+^ and CM-R^−^.

## RESULTS

### Brain-derived RNA is enriched in plasma EVs from patients with cerebral malaria

**Fig. 1.**
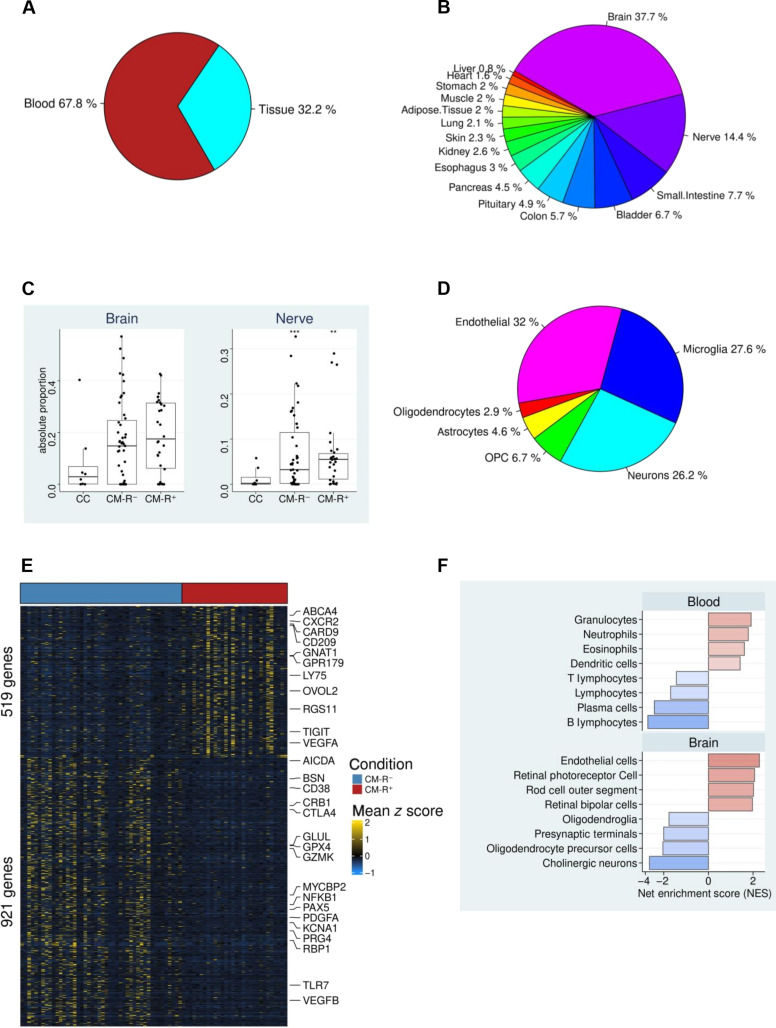
The solid tissue atlas of circulating EV-RNA in cerebral malaria. (**A**) The relative comparison of blood and solid tissue RNA fractions in plasma EVs from patients with cerebral malaria. (**B**) The relative distributions of solid tissue fractions of circulating EV-RNA in cerebral malaria. (**C**) The estimated absolute proportion of RNA expressed by the brain and nerves is higher in retinopathy-positive (CM-R^+^) and negative (CM-R^−^) cerebral malaria compared to community controls (CC). (**D**) Brain cell relative fractions estimated from the plasma EV-RNA data. (**E**) Heatmap clustering of differentially enriched genes between patients with CM-R^+^ and CM-R^−^. (**F**) Top terms associated with genes significantly increased (red) and decreased (blue) in CM-R^+^. The top terms in the blood and brain domains are shown.

The study included 76 children admitted with cerebral malaria at Kilifi County Hospital as previously described (*26*, *27*) (clinical parameters provided in table S1 and their summaries in table S2) and eight community controls (CCs) without *P. falciparum* infection. We purified plasma EVs from all individuals and sequenced their RNA content, then applied support vector regression (*28*, *29*) to deconvolute the composition of solid tissues and brain cells within the EV–RNA sequencing (RNAseq) data (Fig. 1, A to D, and fig. S1A). We found that 32.2% of the plasma EV-RNA isolated from patients with cerebral malaria originated from genes highly expressed in solid tissues (Fig. 1A), and 67.8% of these were from genes highly expressed in whole blood cells (Fig. 1A). Within the solid tissue fraction, the brain predominated (37.7%), followed by peripheral nerves (14.4%) and the small intestines (7.7%) (Fig. 1B and fig. S1A). In addition, the absolute proportion of RNA from transcripts expressed by the brain and peripheral nerves was relatively higher in CM-R^+^ and CM-R^−^ compared to CC (Fig. 1C). Within the brain fraction, RNA from genes highly expressed in brain-endothelial cells (32%), microglia (27.6%), and neurons (26.2%) dominated the plasma EV-transcriptomes, while genes expressed in other brain cell types contributed less than 10% (Fig. 1D). To validate the EV deconvolution approach, we downloaded and deconvoluted plasma EV-RNAseq data (GSE100207) generated from patients with hepatocellular carcinoma (HCC) (*30*). We observed that tissue-derived plasma EV-RNA in patients with HCC originated predominantly from the liver (31.9%) and the adipose tissue (23.5%) (fig. S1, B and C), thus validating the EV-origin deconvolution analysis.

Next, we performed differential feature analysis and identified 1440 significantly altered genes, of which 521 were enriched in CM-R^+^, while 921 were enriched in CM-R^−^ (Fig. 1E and table S3). Cellular enrichment analysis of the 521 genes enriched in CM-R^+^ strongly associated them with granulocytes (neutrophils and eosinophils), endothelial cells, and retinal photoreceptor cells (Fig. 1F). Conversely, the 921 genes enriched in CM-R^−^ were strongly linked to lymphocytes, cholinergic neurons, presynaptic terminals, and oligodendrocyte precursor cells (Fig. 1F).

### Manifold learning of plasma EV-RNA suggests that CM-R^−^ might precede CM-R^+^ in the pathological continuum

**Fig. 2.**
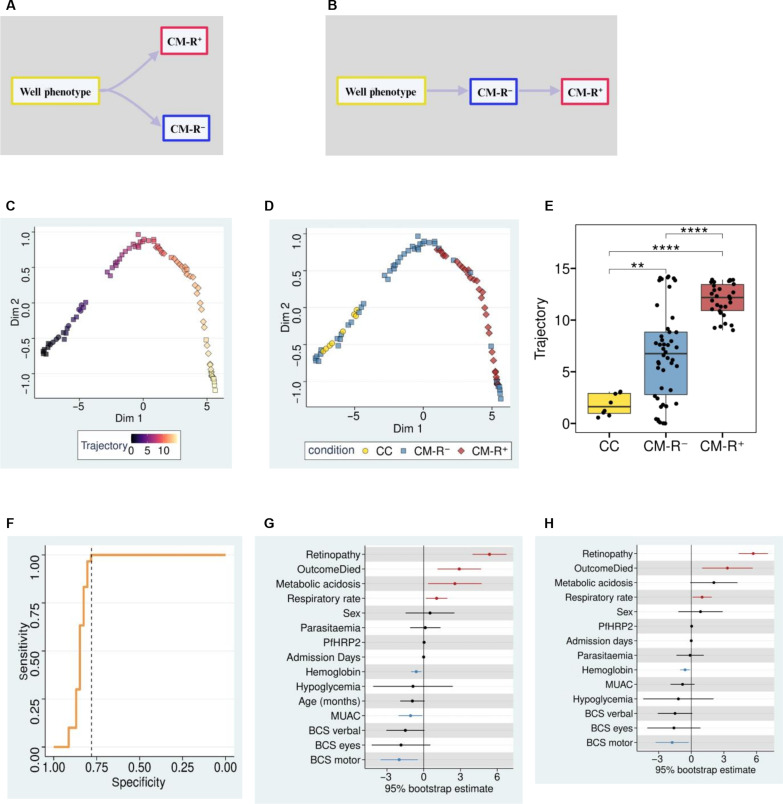
Manifold learning infers disease trajectory from plasma-EV transcriptomes. (**A** and **B**) Schematic representation of two models of disease progression in cerebral malaria; (A) CM-R^−^ is either a disease variant distinct from CM-R^+^ or (B) CM-R^−^ precedes CM-R^+^. (**C**) Scatterplot showing EV-RNA samples colored by disease trajectory. The scatter plot shows cerebral malaria evolves in a single lineage. (**D**) EV-RNA samples colored by retinopathy status, depicting that late-stage trajectory is enriched for the CM-R^+^ sample set. (**E**) Boxplots comparing disease trajectory between CC, CM-R^−^, and CM-R^+^. The inferred disease trajectory is significantly more advanced in CM-R^+^ than in CM-R^−^. (**F**) Receiver operating characteristic (ROC) curve shows that retinopathy is 100% sensitive and 78% specific that cerebral malaria has progressed to late stages of the trajectory. (**G** and **H**) Forest plots showing linear regression results (95% bootstrap estimates) comparing retinopathy and clinical parameters, (G) unadjusted and (H) adjusted for age. Red shows positive correlations, black nonsignificant correlations, and blue negative correlations. The estimated molecular trajectory is concordant with known clinical parameters.

CM-R^−^ has been considered by some studies as a disease distinct from CM-R^+^ that is caused by other etiologies besides *P. falciparum* (Fig. 2A) (*4*, *9*, *14*), whereas other studies have suggested that CM-R^−^ could be a less severe variant of cerebral malaria that has the potential to progress to CM-R^+^ (Fig. 2B) (*15*, *16*). We, therefore, explored whether our plasma EV transcriptomes could be used to resolve cerebral malaria disease progression at the molecular level. We used manifold learning on the EV transcriptomes obtained from the cross-sectionally sampled patients to define the molecular data–driven disease progression model (Fig. 2C), often called trajectory (*23*). The patients with cerebral malaria were ordered based on their similarity in EV-RNA abundance using the CC to locate the starting point, and this order was used to infer the molecular disease trajectory (Fig. 2C). This analysis showed a single disease lineage with samples in later-stages of the trajectory being those from patients with CM-R^+^, while earlier samples primarily originated from CM-R^−^ and CC (Fig. 2D). We then used the Wilcoxon signed-rank test to compare disease trajectory between CM-R^+^, CM-R^−^, and CC and found that disease trajectory was significantly (*P* value <0.001) more advanced in CM-R^+^ compared to CM-R^−^ (Fig. 2E). However, we found no significant difference in disease trajectory between patients with CM-R^+^ with and without retinal hemorrhage (fig. S2). Using receiver operating characteristic (ROC) analysis, we benchmarked the inferred trajectory against retinopathy and found that retinopathy was 100% sensitive and about 78% specific for late-stage cerebral malaria (Fig. 2F). Next, we applied linear regression to assess whether disease trajectory was associated with clinicopathological parameters provided in table S1. Later trajectory was positively associated with retinopathy, metabolic acidosis, in-hospital death, and respiration rate and negatively associated with mid-upper arm circumference (a surrogate for nutritional status), hemoglobin, and Blantyre coma scores (BCS; motor) (Fig. 2G). When we adjusted the regression models for patient age, we noted that retinopathy, in-hospital death, respiratory rate, hemoglobin, and BCS (motor) were all still associated with disease trajectory (Fig. 2H). Unexpectedly, peripheral parasitemia and plasma *Pf*HRP2 were not associated with disease trajectory even after age adjustment, suggesting that disease trajectory in cerebral malaria is not a simple correlate of parasite burden in this cohort of patients (Fig. 2, G and H). To rule out that the estimated trajectory was not due to the combination of CM-R^−^ and CM-R^+^ in the same analysis, we inferred the trajectory separately for each group and found that separate trajectories were strongly correlated to the combined trajectory (fig. S3). These observations imply that the calculated trajectory is an accurate proxy for disease progression and suggests that CM-R^−^ could be an early-stage disease variant of cerebral malaria that precedes CM-R^+^.

### Plasma EV transcriptomes change as a function of disease trajectory

**Fig. 3.**
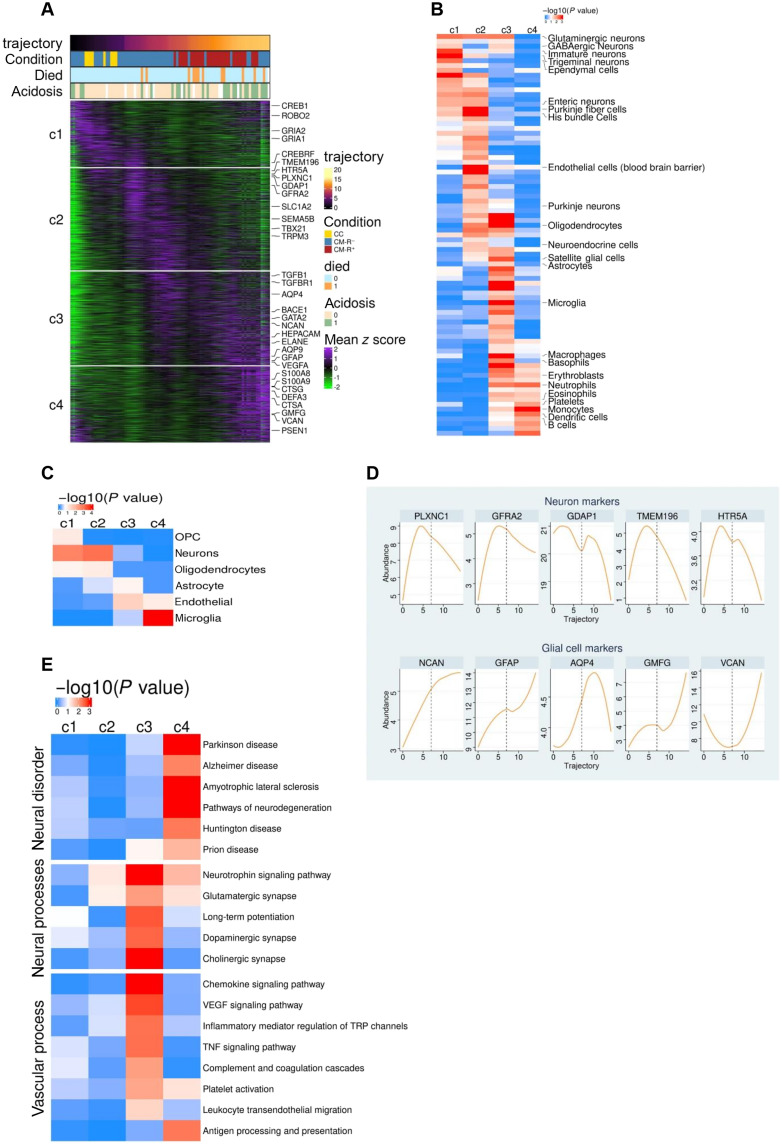
Brain-derived plasma EV-RNA varies over disease trajectory. (**A**) Phaseogram showing the variation of plasma EV-RNA as a function of disease trajectory. The transcripts are clustered along the disease trajectory, revealing four clusters, c1 to c4. (**B**) Enrichment analysis using PanglaoDB cell markers (*31*) shows that early trajectory clusters (c1 and c2) are enriched for neuronal markers, while late-trajectory clusters are enriched for glial (oligodendrocyte, astrocyte, and microglia) and immune cells. (**C**) Enrichment analysis using Darmanis brain cell markers (*32*) also shows that cerebral malaria is characterized by decreased and increased neuronal and glial gene expression respectively. (**D**) Representative EV-RNA profiles of neuronal and glial cell markers. (**E**) KEGG enrichment analysis results showing that cluster 3 is enriched for transcripts belonging to vascular processes and synaptic-related neural functions, while cluster 4 is enriched for transcripts linked to pathways of neurodegeneration.

To dissect global transcriptional patterns over disease trajectory, we fitted nonlinear regression models to the gene profiles and found that 70% of the total EV transcripts (7438 of 10,150) were significantly altered as a function of disease trajectory (nominal *P* value <0.05) (table S4). We then ordered the samples and genes over the trajectory to construct a phaseogram using the significantly altered transcripts, and clustering analysis using *k*-means identified four gene clusters, which we named c1 to c4 (Fig. 3A and table S4). Subsequently, we used Fisher’s exact test to analyze the gene overlap between the clusters and reference lists of published cell type–specific markers (*31*, *32*). Early-trajectory gene clusters (c1 and c2), which broadly decreased with disease trajectory, were enriched for neuronal gene sets, while the late ones (c3 and c4), which increased with disease trajectory, were enriched for glial (astrocytes and microglia) and blood cell (notably neutrophils and erythroblasts) gene sets (Fig. 3, B and C, and tables S5 and S6). We exemplify the above observations using smoothed curves of five neuronal markers, including serotonin receptor (HTR5A), glial-derived neurotrophic factor receptor alpha 2 (GFRA2), and ganglioside-induced differentiation-associated protein 1 (GDAP1) and five glial cell markers, the most notable being markers of reactive gliosis, neurocan (NCAN) (*33*) and glial fibrillary acidic protein (GFAP) (*34*), and the astrocytic water channel aquaporin 4 (*35*) (Fig. 3D). These results insinuate that the molecular trajectory of cerebral malaria is characterized by a smooth transcriptional cascade of declining neuronal transcripts and a progressive increase in glial and immune cell transcripts, which can be studied via EVs circulating in peripheral blood.

We proceeded to perform enrichment analysis using the Kyoto Encyclopedia of Genes and Genomes (KEGG) gene sets (*36*) to determine whether transcripts enriched in late-trajectory clusters (c3 and c4) belong to biological pathways that could be associated with cerebral malaria pathogenesis. Although there was a global decrease in neuronal RNA in EVs (Fig. 3, B and C), neuronal processes linked to synaptic plasticity (long-term potentiation, glutamatergic synapse, and neurotrophin signaling) (*37*–*39*) were enriched in cluster 3 (Fig. 3E). In addition, vascular processes [tumor necrosis factor signaling, vascular endothelial growth factor (VEGF) signaling, platelet activation, and the complement cascade] were enriched in cluster 3, while cluster 4 was enriched for genes implicated in age-related disorders such as Parkinson’s disease, amyotrophic lateral sclerosis, Huntington’s disease, and Alzheimer’s diseases (Fig. 3E and table S7). Together, our data show that it is feasible to study noninvasively the pathological processes that drive infectious encephalopathies, such as cerebral malaria, by analyzing the biological contents of circulating EVs.

### Disease trajectory resolves the heterogeneity within CM-R^−^ by identifying three subgroups

**Fig. 4.**
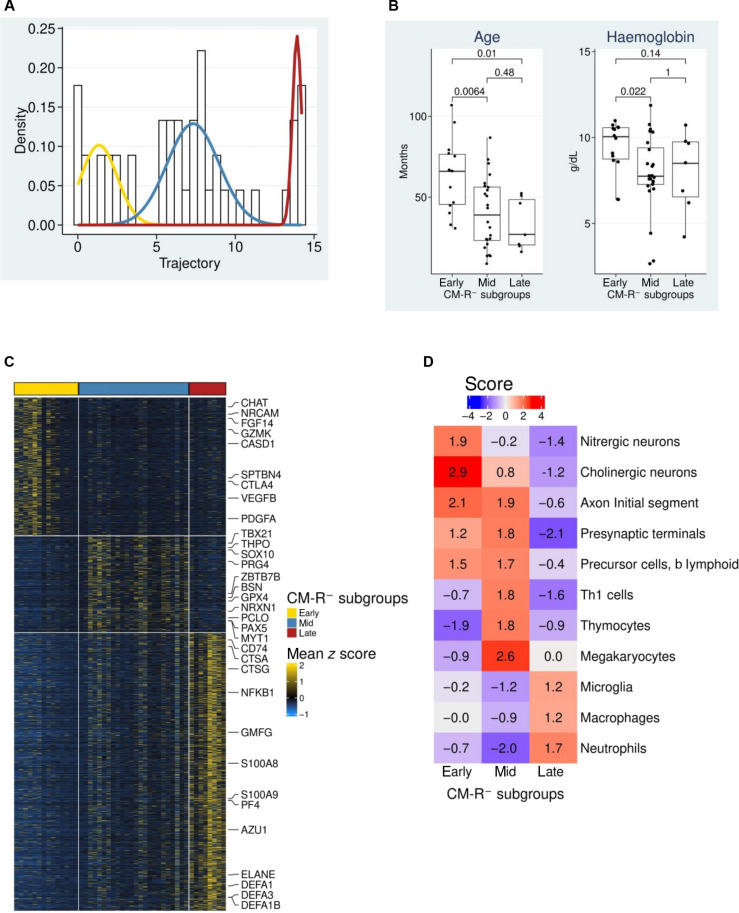
Disease trajectory stratifies patients with CM-R^−^ into three subgroups. (**A**) Gaussian mixture modeling (GMM) shows that the patients with CM-R^−^ cluster optimally into three subgroups: early trajectory (yellow), mid-trajectory (blue), and late trajectory (red). (**B**) Patients in the early CM-R^−^ subgroup tended to be older and less anemic than those in the mid- and late-trajectory subgroups. (**C**) Supervised heatmap clustering showing differentially altered genes between the CM-R^−^ subgroups. (**D**) Top terms from the domains of blood and brain that are enriched in at least one CM-R^−^ subgroup.

The disease trajectory revealed notable intragroup heterogeneity within patients with CM-R^−^ with a subset clustering within the patients with CM-R^+^ (Fig. 2D). Thus, we proceeded to evaluate the distribution of the inferred trajectory using Gaussian mixture models (GMM) and identified three as the optimal number of subgroups of the patients with CM-R^−^ (Fig. 4A). Among the patients with CM-R^−^, 14 (30.4%), 28 (52.2%), and 8 (17.4%) fall within early-, mid- and late-trajectory subgroups, respectively (Fig. 4A). We then compared the clinical parameters (table S1) between the three trajectory subgroups and noticed that children in the early-trajectory subgroup tended to be older and less anemic compared to those in mid- and late-trajectory subgroups (Fig. 4B). Next, we performed differential analysis and identified 3386 differentially altered genes between the three CM-R^−^ trajectory subgroups (Fig. 4C and table S8). We found that the neuronal RNA in plasma EVs was lower in the late subgroup compared to the early- and mid-subgroups of CM-R^−^. Conversely, relative to the early-trajectory CM-R^−^ subgroups, which clustered with the CCs (Fig. 2D), our enrichment analysis showed that markers associated with T-helper cells (e.g., TBX21), thymocytes (e.g., ZBTB7B), and megakaryotes (e.g., THPO) were enriched in the mid-trajectory CM-R^−^ subgroup (Fig.4D), while markers associated with myeloid cells such as macrophages/microglia (e.g., CD68 and CX3CR1) and neutrophils (e.g., ELANE, AZU1, and CTSG) were enriched in the late-trajectory CM-R^−^ subgroup (Fig. 4D). These molecular differences in plasma EV-RNA from the three trajectory CM-R^−^ subgroups indicate that CM-R^−^ is a heterogeneous group of cerebral malaria patients at different stages of the pathological spectrum.

## DISCUSSION

Cerebral malaria is a complication of *P. falciparum* characterized by impaired consciousness, among other neurological complications (*1*, *40*). However, despite extensive research, the pathological processes by which malaria parasites cause cerebral malaria (*41*, *42*) are poorly defined. In this study, we hypothesized that the RNA content of circulating EVs could be used to study neuropathological processes during cerebral malaria, specifically the transcriptional profiles of the cerebral tissues. We show that the RNA content of circulating EVs reflects biological processes that occur during cerebral malaria and could be used as a noninvasive means to study disease mechanisms and identify diagnostic biomarkers.

Our results showed that after blood, brain cells predominated as the source of circulating EV-RNAs in patients with cerebral malaria. Our trajectory inference analysis showed a single lineage of disease progression with late-stage trajectory enriched for CM-R^+^ samples. This could imply that CM-R^−^ might be a less severe cerebral malaria disease variant that precedes CM-R^+^, aligning with the down-regulation of markers of lymphoid cells (e.g., B cells) and increased markers of myeloid cells (e.g., neutrophils) in CM-R^+^. Such an immune profile has been associated with unfavorable outcomes in several diseases (*43*). In addition, a recent neuroimaging study identified signatures of cerebral malaria in noncomatose adults (*44*), which is consistent with the continuum nature of disease progression. Other recent data (*15*, *16*, *45*) also challenged the concept that CM-R^−^ represents encephalopathies of other etiologies besides *P. falciparum* (*4*, *9*, *14*). Consistently, the inferred trajectory was also positively associated with other malaria clinicopathological parameters of severity, such as in-hospital death, anemia, and depth of coma, reinforcing the hypothesis that disease trajectory, as a latent variable calculated from the EV-RNAseq data, represents the pathological continuum of cerebral malaria.

We observed declining neuronal transcript levels during the late stages of the disease trajectory, which coincides with increased glial (astroglia and microglia) transcripts. The increase in glial transcripts likely indicates progressive activation of astrocytes and microglia, as observed previously in experimental (*46*–*48*) and human cerebral malaria (*49*–*51*). Astrocytes form part of the neurovascular unit, interact with neurons and the vascular system (*52*), and thus respond to neuronal and vascular stress signals. The astroglia cell response is usually marked by increased expression of protein constituents of astrocyte intermediate filaments, including GFAP and NCAN (*33*, *34*). We observed that the corresponding RNA from GFAP and NCAN in plasma EVs from patients with cerebral malaria increased with disease trajectory. Although our data showing neuronal decline and increased glial gene expression are consistent with the trend observed in neurodegenerative diseases (*23*), neurological impairment in cerebral malaria is usually reversible, except in the minority with severe disease. This suggests that gliosis in cerebral malaria indicates an early response to vascular injury (*53*), and the net decrease in neuronal transcripts in circulating EVs may indicate gradual neuronal hypofunction with disease progression rather than neuronal death (*54*), except in extremely severe cases (*41*) as the body temporarily suspends most of the homeostatic functions to prioritize adaptive prosurvival functions such as those promoted by synaptic glutamatergic and neurotrophins signaling (*38*, *55*).

Gaussian mixture model on disease trajectory identified three CM-R^−^ subgroups with distinct molecular profiles of EV-RNA. The last of the three overlapped with CM-R^+^, suggesting that these patients are likely “true” cerebral malaria cases missed by the ophthalmological assessment (*56*). Three key findings on the trajectory of CM-R^−^ emerged from this complimentary analysis: (i) decline of neuronal function with disease trajectory as indicated by reduced neuronal gene transcript such as those of cholinergic neurons, (ii) lymphocyte-mediated immune response such as B and T helper immune response peaks during mid-trajectory, and (iii) up-regulation of myeloid cell markers including neutrophils, a common phenomenon in CM-R^+^ (*57*), occur during advanced stages of CM-R^−^. These observations could indicate a pathological spectrum where CM-R^−^ is a step in the progression to CM-R^+^ or an overlap between CM-R^−^ and CM-R^+^.

A limitation of our study is that cerebral malaria disease progression in children may not be generalized to adults. Specifically, adult studies on cerebral malaria (*12*, *13*) have found lower retinopathy rates than those found in pediatric studies, and retinopathy was observed in uncomplicated malaria (*6*, *9*, *11*, *14*). Twenty one of the 30 CM-R^+^ had a retinal hemorrhage, thus limiting comparison between the retinopathy features. Despite the inferred trajectory being highly concordant with the clinical severity measures, it can only be proposed as a notional derivative. The ethical imperative to start treatment precludes informative longitudinal sampling of severe disease. Therefore, real-world patient admission datasets are predominantly cross-sectional, and analytical approaches to infer disease trajectory to understand the molecular events leading to disease progression are increasingly being used for similar datasets (*58*, *59*). Although our data might suggest that CM-R^−^ and CM-R^+^ are on a continuum, the small sample size may have limited our ability to capture the true variation of cerebral malaria disease trajectories. In addition to increasing the sample size, including samples from patients with uncomplicated malaria and other severe phenotypes would have provided additional scope to test these hypotheses. Furthermore, there are no in vivo data comparing the biological content of plasma EVs to the metabolic and physiological status of brain tissues during cerebral malaria, and future studies comparing EV contents with signatures from brain postmortem samples could be revealing.

Taking our findings together, we speculate that reduced microcirculatory flow resulting from parasite sequestration in the brain (*4*) leads to suboptimal brain perfusion (*60*–*62*) and neuronal hypofunction, evidenced by falling neuronal transcript levels. This is associated with a progressive increase in glial cell activity (*52*) (evidenced by a progressive increase in glial transcripts), as well as brain adaptive processes such as neurotrophin signaling (*63*), long-term potentiation (*38*), and glutamatergic signaling (*64*, *65*). Together with vascular signaling (VEGFA-VEGFR2) (*66*–*72*), platelet activation and coagulation (*73*, *74*), and neutrophil activation (*57*), these are adaptive processes during the late stages of cerebral malaria (*38*, *75*), which may turn maladaptive and pathological (*38*, *75*). For example, VEGFA-VEGFR2 signaling, acting on the vascular smooth muscle and endothelial cells (the effectors), induces vasodilation. However, vasodilation occurs at the expense of vascular integrity, which may lead to pathological disruption of the blood-brain barrier (*70*, *76*), hemorrhage (*6*, *77*, *78*), and extravasation of plasma content into the brain parenchyma (resulting in vasogenic edema) (*7*, *72*, *79*–*81*). Similarly, overexcitation of synaptic glutamatergic signaling may lead to pathological conditions such as epileptic seizures and neuronal dysfunction (*65*, *82*).

In conclusion, we show that the contents of circulating EVs can be used to study the brain in children with cerebral malaria. We demonstrate that the molecular sequence of neurovascular events in pediatric cerebral malaria is accessible antemortem via EVs despite the inaccessibility of brain tissues to direct sampling. This will allow a more complete study of the pathogenesis of the illness, identification of biomarkers to predict disease progression, and design of therapeutic interventions.

## METHODS

### Samples and design

The EV-RNAseq data were generated from 76 archived plasma samples from children with cerebral malaria who had been assessed for retinopathy and eight community-control adults without *P. falciparum* infection. The Scientific Ethics Review Unit (SERU) of the Kenya Medical Research Institute (KEMRI) under the protocol KEMRI/SERU/3149 provided ethical approval of the study. Written consent was provided by the parents or guardians of the children who donated the plasma samples. The subset of patients included in this study represented the whole cohort, as the proportion of CM-R^+^ (30 of 76; 39%) and CM-R^−^ (46 of 76; 61%) was largely similar to those documented in the original studies (*26*, *27*). Among the CM-R^+^ samples, 21 of 30 were obtained from patients with retinal microhemorrhage. Sample size was pragmatically determined based on available samples, clinical data, and resources.

### Comparison of the clinical parameters of patients with CM-R^+^ and CM-R^−^

The values presented in table S2 were calculated as follows: BCS, in-hospital death, hypoglycemia, and metabolic acidosis were treated as categorical variables, and the χ^2^ test was adopted to compare differences between CM-R^−^ and CM-R^+^. The remaining variables were treated as continuous and Shapiro-Wilk test showed nonnormal distribution for all. Therefore, the Wilcoxon signed-rank test was used to compare the differences in the medians.

### Isolation of EVs from plasma and RNA extraction

Two hundred microliters of plasma was diluted in 13.5 ml of phosphate-buffered saline (PBS) and passed through a 0.22-μm filter. The filtrate was transferred into new ultracentrifuge tubes and spun at 150,000*g* for 2 hours at 4°C. The supernatant was discarded, while the pellet was resuspended in 300 μl of PBS and treated with ribonuclease A at 37°C to digest nonvesicular RNA. After 15 min, the mixture was transferred to 13.5-ml ultracentrifuge tubes (Beckman). The tubes were filled using PBS and ultracentrifuged at 150,000*g* for 2 hours at 4°C. The final pellets were digested using 250 μl of RNA lysis buffer (Bioline) and kept at −80°C until when required. RNA was isolated using the Isolate II RNA Min Kit (Bioline), following the manufacturer’s instructions. Bead-assisted flow cytometry using antibodies to EV markers CD63 and CD9 was used to validate the EV isolation protocol (*83*).

### Library preparation from plasma EV-RNA

The deoxyuridine triphosphate (dUTP) protocol developed by Chappell *et al.* (*84*) was adopted to prepare the cDNA libraries for sequencing as we previously did (*83*). Briefly, total EV-RNA is used to generate the first strand. Before second strand synthesis, the samples were cleaned using RNAcleanXP beads to remove traces of dNTPs. During the synthesis of the second strand, 3′-deoxythymidine 5′-triphosphate was replaced with dUTP. Double-stranded cDNA was then enzymatically shredded and ligated to NEXTflex adapters. The cDNA was treated with uracil glycosylase, which digests dUTPs to make the libraries strand-specific and amplified in 15 cycles to increase yield. Sequencing was done in two batches: (i) using the Hiseq 4000 genome analyzer at the Wellcome Sanger Institute, UK and (ii) using the NextSeq 550 genome analyzer at the International Livestock Research Institute, Kenya.

### Normalization of RNAseq data

Batch effects were assessed using principal component analysis (fig. S4). RNAseq fastq files were quality checked, and transcript read estimates were obtained by aligning the data to the human transcriptome using Kallisto (*85*). The count data were normalized by gene length and sequencing depth, converted to counts per million units, and used as input for all downstream analyses.

### Deconvolution of EV-RNA data

Support vector regression was used to estimate the RNA fractions of solid tissue and brain cell–specific RNA. The solid tissue signature matrix used is publically available (*29*), while the brain cell signature matrix was constructed from the Darmanis brain cell data (*32*, *86*). The blood-tissue matrix was generated by determining marker genes between solid tissues and blood using the Human Protein Atlas tissue RNAseq data (*87*). The Seurat tool was used to find the tissue-specific markers.

### Differential feature analysis between CM-R*^+^* and CM-R^−^

Differential expression analysis was performed using the edgeR Bioconductor package. The likelihood ratio test was used to test for differential significance, and correction for multiple comparisons was performed using the Benjamin-Hochberg (BH) procedure. The cutoff for significance was set at absolute (logFC) >1 and false discovery rate (FDR) <5%. The Literature Lab gene enrichment tool was used to perform the enrichment analysis.

### Estimation of cerebral malaria disease trajectory

We used deep learning to infer the trajectory of the EV transcriptome samples using a method implemented in the PhenoPath R package (*88*) and visualized using manifold learning. PhenoPath is a tool that employs Bayesian statistics to model the latent expression of each sample. Bootstrapped linear regression models were used to relate pseudotime to clinical parameters of severe malaria. The bootstrapping approach was adopted to correct biases when assumptions of linear regression are not fully met. Harmonic regression models were fitted to determine the EV-RNAs altered as a function of the inferred disease trajectory, and a nominal *P* value <0.05 was used as the cutoff for significance. Linear regression and ROC were used to compare disease trajectory to retinopathy and other clinicopathological parameters, while Spearman’s rank correlation was applied to determine the association of brain cell EV-RNA fractions with disease trajectory. The phaseogram of disease trajectory was constructed using ComplexHeatmap and subdivided into four clusters using *k*-means (*89*). The overlap between the four clusters and a published reference list of brain cell–specific markers (*31*, *32*) was tested using Fisher’s exact test. KEGG analysis was also performed using Fisher’s exact test. Visualization was performed using heatmaps in R, where neuronal, glial, and vascular gene sets previously linked to cerebral malaria were highlighted.

### Identification of CM-R^−^ subgroups

Gaussian mixture modeling of the calculated trajectory, provided by the mixtools R package, was used to stratify CM-R^−^ samples into three subgroups. An analysis of variance (ANOVA)–like differential expression analysis was performed in edgeR to identify differentially enriched genes between the three CM-R^−^ subgroups. Correction for multiple testing was performed using the BH procedure, and only genes reaching an FDR threshold of less than 5% were considered significant. Enrichment analysis was performed using the Literature Lab gene enrichment tool.

## Supplementary Material

20240816-1

## Notes

**Correction (10 September 2024):** The published version of Fig. 3 was not the most recent version, and the error was only recently brought to the attention of the Editorial Office. The labels “psuedotime” were changed to “trajectory” in Fig. 3, A and D. The XML and PDF versions of the paper have been updated.
