# Supplementary material for: The mRNA content of plasma extracellular vesicles provides a window into molecular processes in the brain during cerebral malaria
Source: Sci Adv. 2024 Aug 16;10(33):eadl2256. doi: 10.1126/sciadv.adl2256 (PMC11328904; doi:10.1126/sciadv.adl2256)
Supplement: Supplementary file 2 — Figs. S1 to S4 Legends for tables S1 to S8 [file sciadv.adl2256_sm.pdf]

Science Advances
AAAS

Supplementary Materials for

The mRNA content of plasma extracellular vesicles provides a window into molecular processes in the brain during cerebral malaria

Mwikali Kioko *et al.*

Corresponding author: Abdirahman I. Abdi, aabdi@kemri-wellcome.org

Sci. Adv. **10**, eadl2256 (2024)
DOI: 10.1126/sciadv.adl2256

The PDF file includes:

Figs. S1 to S4
Legends for tables S1 to S8

Other Supplementary Material for this manuscript includes the following:

Tables S1 to S8

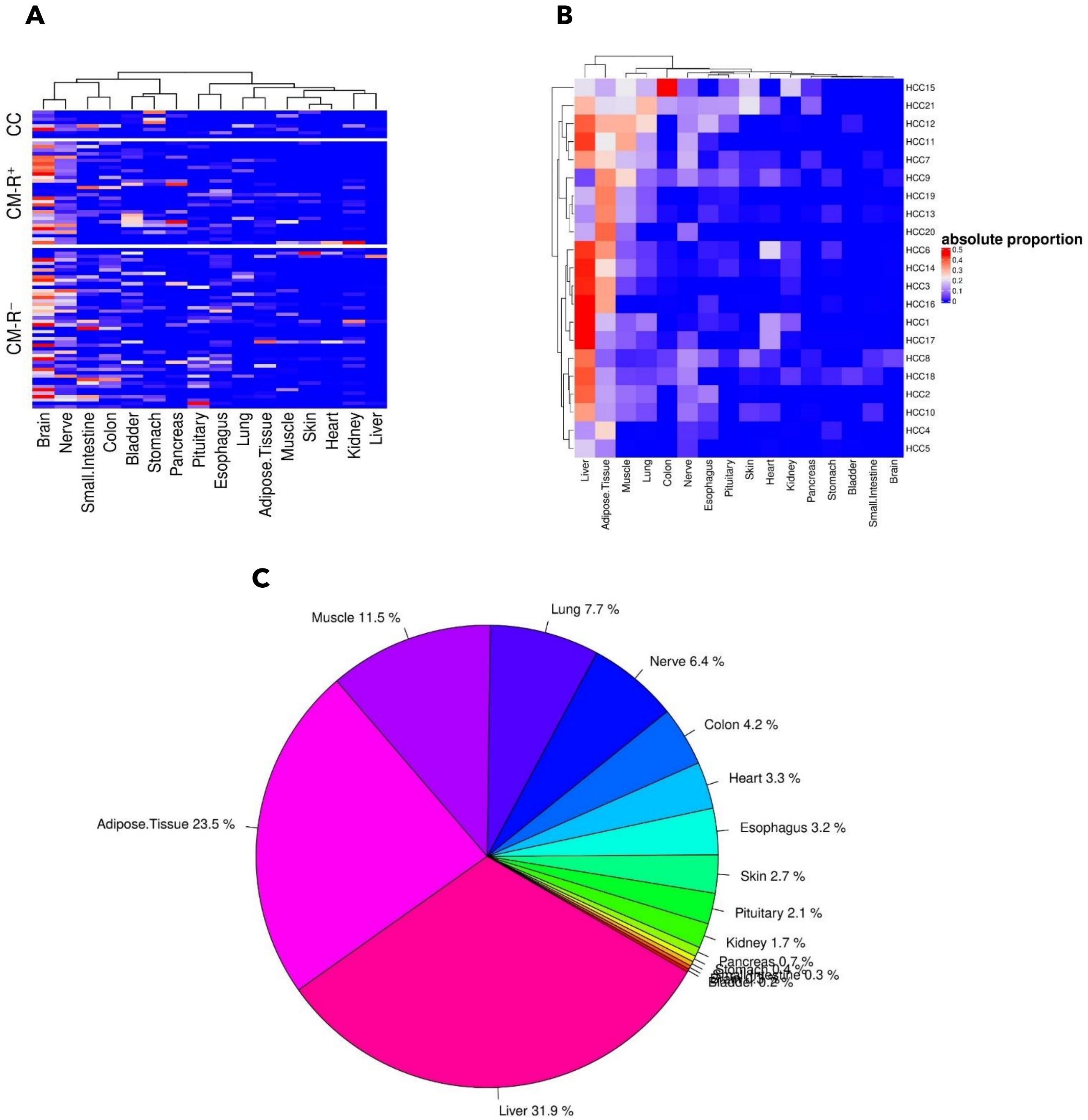

Fig. S1 EV-deconvolution and validation using plasma EV-RNAseq (GSE100207) obtained from hepatocellular carcinorma patients (HCC). A Absolute RNA fractions of solid tissues estimated from the EV-RNA from retinopathy positive ($CM\text{-}R^{+}$) and negative ($CM\text{-}R^{-}$) cerebral malaria compared to community controls (CC) **B** Absolute fractions of solid tissues estimated from the EV-RNA from HCC patients. **C** Relative distribution of solid tissue factions estimated from the HCC EV-RNA. As expected, the highest fraction of RNA in circulating EVs from HCC patients is expressed in the liver.

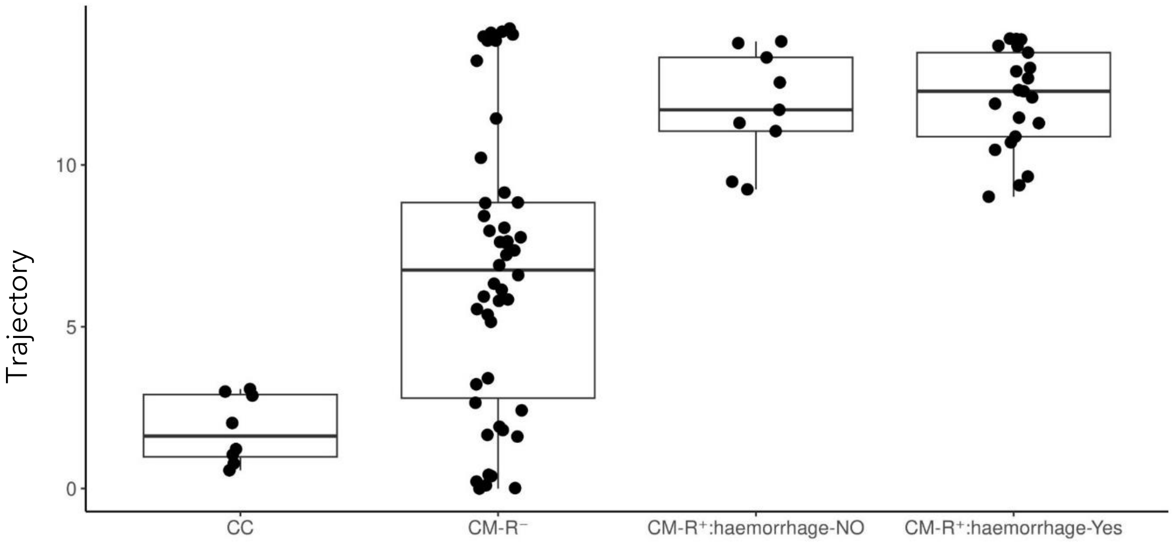

Fig S2: Disease trajectory was not different between retinopathy positive patients with or without retinal haemorrhage.

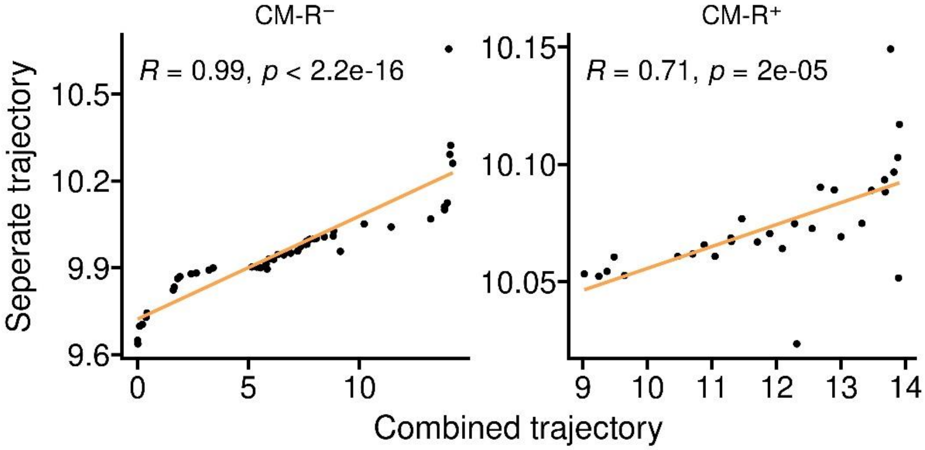

Fig S3: Disease trajectories inferred from retinopathy-negative and positive samples separately are highly correlated to the combined trajectory

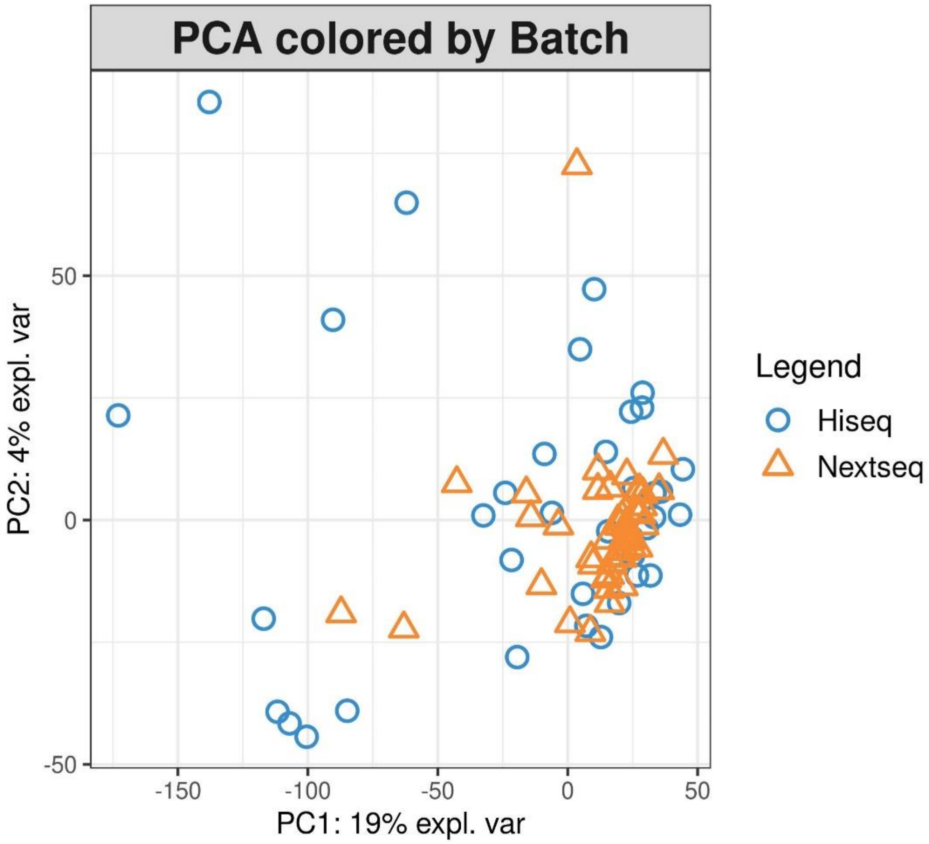

Fig S4: PCA analysis showing that samples do not cluster based on the sequencing platform. Each dot represents a sample.

Data Table S1 (separate file) – Clinicopathological parameters: Excel spreadsheet with patient's clinicopathological parameters.

Data Table S2 (separate file) – Summary of samples by malaria retinopathy status: Excel sheet comparing clinical parameters between retinopathy-positive and negative cerebral malaria

Data Table S3 (separate file) – Differential expression analysis: Excel sheet with Differential expression analysis results comparing retinopathy-positive to negative cerebral malaria

Data Table S4 (separate file) – Non-linear regression analysis: Excel sheet with regression analysis results showing transcript change as a function of disease trajectory.

Data Table S5 (separate file) – Enrichment analysis: Results obtained from enrichment analysis using PanglaoDB gene sets.

Data Table S6 (separate file) – Enrichment analysis: Results obtained from enrichment analysis using Darmanis brain Single-cell RNAseq markers.

Data Table S7 (separate file) – Enrichment analysis: Results obtained from enrichment analysis using KEGG genesets.

Data Table S8 (separate file) – Differential expression analysis: Excel sheet with Differential expression analysis results comparing retinopathy-negative cerebral malaria disease trajectory subgroups
